# Case Report: SARS-CoV-2 Associated Acute Interstitial Nephritis in an Adolescent

**DOI:** 10.3389/fped.2022.861539

**Published:** 2022-04-14

**Authors:** Karolis Azukaitis, Justinas Besusparis, Arvydas Laurinavicius, Augustina Jankauskiene

**Affiliations:** ^1^Clinic of Pediatrics, Institute of Clinical Medicine, Faculty of Medicine, Vilnius University, Vilnius, Lithuania; ^2^Institute of Biomedical Sciences, Faculty of Medicine, Vilnius University, Vilnius, Lithuania

**Keywords:** acute interstitial nephritis, children, coronavirus, COVID-19, pediatric, SARS-CoV-2

## Abstract

Acute interstitial nephritis (AIN) has been recently recognized as one of the infrequent kidney involvement phenotypes among adult patients with severe acute respiratory syndrome coronavirus 2 (SARS-CoV-2) infection. Although SARS-CoV-2 associated intrinsic kidney disease has been scarcely reported in children, only one case of AIN temporally associated with the infection has been described in the pediatric population so far. We presented a case of a 12-year old boy who presented with fatigue, anorexia, and polydipsia following an RT-PCR that confirmed SARS-CoV-2 infection seven weeks prior to admission. Initial workup revealed increased serum creatinine (235 μmol/L), glucosuria, low-molecular-weight proteinuria, mild leukocyturia, and microhematuria with hyaline and granular casts on microscopy. Antibodies against the SARS-CoV-2 S protein receptor-binding domain confirmed prior infection with high titers. Kidney biopsy showed diffuse active interstitial nephritis with negative immunofluorescence and positive immunohistochemistry for SARS-CoV-2 in the inflammatory cells within the interstitium. Electron microscopy revealed several SARS-CoV-2-like particles. Kidney function continued to deteriorate despite several days of supportive therapy only (peak serum creatinine 272 μmol/L); thus, treatment with methylprednisolone pulse-dose therapy was initiated and was followed by oral prednisolone with gradual tapering. Kidney function completely recovered after 3 weeks post-discharge and remained normal after 11 weeks of follow-up (last estimated glomerular filtration rate 106 ml/min/1.73 m^2^) with only residual microhematuria. Our case adds to the emerging evidence of SARS-CoV-2 as a potential etiological agent of AIN in children and also suggests that interstitial kidney injury may result from secondary inflammatory damage. Epidemiological history, serologic testing, and SARS-CoV-2 detection in biopsy should be considered in the work-up of children with AIN of unknown etiology.

## Introduction

Severe acute respiratory syndrome coronavirus-2 (SARS-CoV-2) infection has been associated with a wide range of kidney involvement, encompassing tubular, vascular, glomerular, and interstitial injury (1). The mechanisms of SARS-CoV-2 associated kidney disease appear to be heterogeneous, multifactorial, and are not yet fully elucidated with conflicting data suggesting possible direct kidney infection by the virus (1, 2). Although the data in the pediatric population remains scarce, cases of intrinsic kidney pathology have been reported in children with SARS-CoV-2 infection and include new-onset or relapsed nephrotic syndrome, glomerulonephritis, hemolytic uremic syndrome, and kidney-transplant involvement (3).

Acute interstitial nephritis (AIN) has been recently recognized as a distinct phenotype of kidney injury in adults with COVID-19 but evidence about its underlying mechanisms and the clinical course remains limited (4). Recently, the first case of AIN following SARS-CoV-2 has been described in a pediatric patient (5). AIN may represent an underrecognized kidney manifestation in the pediatric population where the unique courses of COVID-19, including delayed complications related to hyperinflammation and dysregulated immune response, are encountered (6).

We described a case of an adolescent boy who developed AIN following COVID-19, with histological evidence suggesting the presence of SARS-CoV-2 in the kidney tissue.

## Case Description

The patient was referred to our tertiary care center due to general malaise, loss of appetite, increased thirst, and a significant increase in serum creatinine. The patient was an otherwise healthy 12-year-old boy who had a RT-PCR confirmed SARS-CoV-2 infection with mild respiratory symptoms 7 weeks before the admission. The aforementioned clinical symptoms started ~1 week after the infection. The patient had no history of non-steroid anti-inflammatory drugs or other medication use prior to the development of symptoms or admission to the hospital. On admission, the patient was afebrile, had normal blood pressure (BP) and clinical examination was otherwise unremarkable.

**Figure 1:**
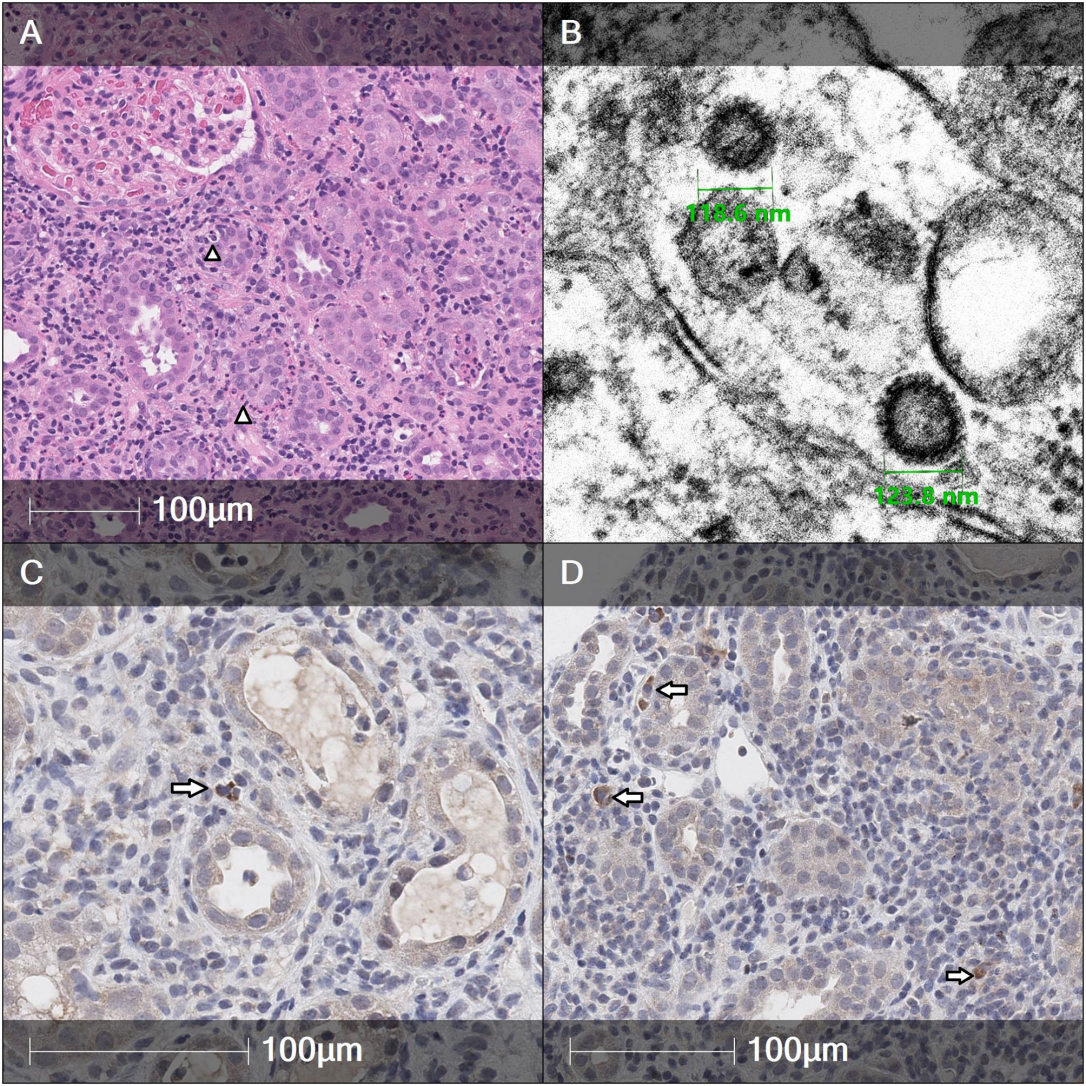
Histological features of kidney biopsy showing diffuse active tubulointerstitial nephritis. **(A)** H&E slide. Marked lymphocytic infiltration in the interstitium with an admixture of neutrophils and plasma cells. Focal features of acute tubulitis are also present (arrowheads). Glomeruli reveal no apparent histological changes. **(B)** Electron microscopy sample is taken from interstitium between renal tubules. Few virus-like particles in size of ~120 nm with barely visible spikes are present intracellularly. Cross-sections of the particles contain interior electron-dense black dots which could be interpreted as helical nucleocapsid (lower magnification is available in Supplementary Figure 1). **(C,D)** Immunohistochemistry (IHC) for Anti-SARS-CoV-2 spike glycoprotein highlighted positive scattered immune cells in the interstitium of renal parenchyma (arrows). Rabbit polyclonal antibody against severe acute respiratory syndrome coronavirus 2 (SARS-CoV-2) spike glycoprotein (ab272504, Abcam) was applied. Lung tissue of a deceased COVID-19-infected patient was used as a positive tissue control and a negative reagent control was performed on a consecutive biopsy tissue section to ensure sensitivity and specificity of the IHC test.

His blood tests revealed severe impairment of kidney function (serum creatinine 235 μmol/ml), high erythrocyte-sedimentation rate, anemia, thrombocytosis, and increased C-reactive protein. Urine tests showed glucosuria, low-molecular-weight proteinuria, and microhematuria, mild leukocyturia with hyaline and granular casts on microscopy. Initial laboratory results are presented in Table 1. Antibodies against SARS-CoV-2 S protein receptor-binding domain confirmed prior infection with high titers (453 BAU/ml). Kidney ultrasound showed bilateral parenchymal thickening and hyperechogenicity with normal corticomedullary differentiation. AIN was suspected and the patient underwent a kidney biopsy which revealed diffuse active interstitial nephritis with negative immunofluorescence and positive immunohistochemistry (IHC) staining for SARS-CoV-2 in interstitial cells, and SARS-CoV-2-like particles by electron microscopy (EM) (Figure 1, lower magnification available in Supplementary Figure 1). Positive and negative controls used for IHC staining (including SARS-CoV-2 positive and negative lung tissue, a reagent with the omission of the primary antibody, and kidney tissue from a patient with AIN diagnosed in the pre-COVID-19 era) are presented in Supplementary Figures 2–4.

**Table 1:** Laboratory findings at diagnostic work-up at the time of admission.

**Diagnostic test**	**Result**
White blood cell count (10^9^)	10.29
Serum hemoglobin (g/dL)	9.8
Platelet count (10^9^)	517
Serum creatinine (μmol/L)	235
eGFR (ml/min/1.73 m^2^)	23.0
Urea (mmol/L)	11.8
Erythrocyte sedimentation rate (mm/h)	120
C-reactive protein (mg/L)	49.6
**Urine tests**	
Proteinuria (g/L)	1.5
Glucosuria	3+
Hematuria	3+
Leukocyturia	1+
Urine microscopy	Granular casts, hyaline casts
Urine protein electrophoresis	Albumin−16.8 %; globulins: alpha 1−42.7 %, alpha 2−16.6 %, beta−9.2 %, gamma−14.7 %
**Diagnostic work-up**	
C3 (g/L)	Normal (1.53)
C4 (g/L)	Normal (0.37)
Anti-CMV antibodies	Negative
Anti-EBV antibodies	Negative
Anti-SARS-CoV-2 S protein receptor binding domain antibodies (BAU/mL)	Increased (453.3)
Serum calcium (mmol/L)	Normal (2.58)
Ophtalmological examination	Normal

The complement levels of the patient were normal and no evidence of recent Epstein-Barr (EBV) or cytomegalovirus (CMV) infections was found by serologic testing. Additional workup that included ophthalmological evaluation for uveitis, anti-nuclear antibodies, and total serum calcium levels were all normal (Table 1).

**Figure 2:**
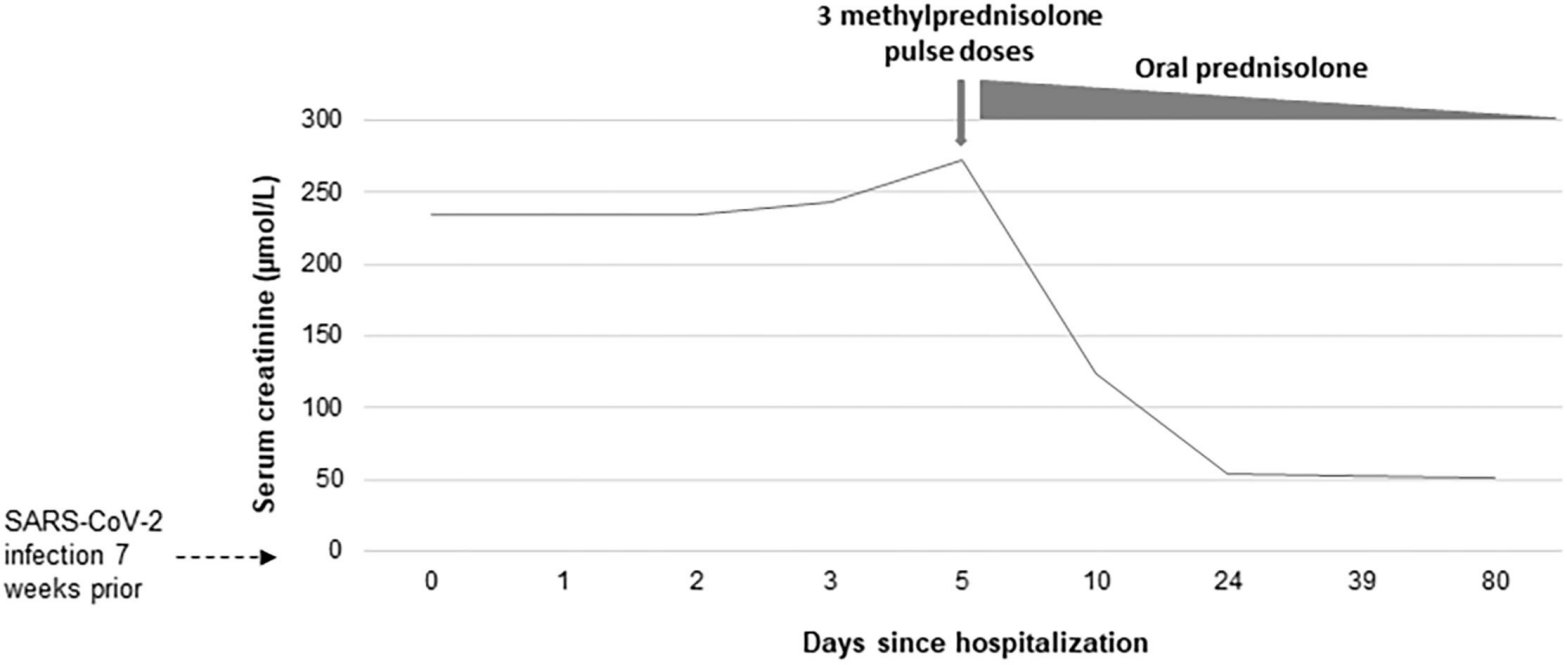
Time course of serum creatinine since the admission to the hospital.

Kidney function continued to deteriorate despite several days of the watch and wait period with supportive therapy only (peak serum creatinine 272 μmol/L), and thus the boy was treated with methylprednisolone pulse-dose therapy (three doses of 1,000 mg). Laboratory testing immediately after treatment revealed significant improvement in kidney function (serum creatinine 124 μmol/L), increase in hemoglobin levels (11.3 g/dL) and decrease in proteinuria (1.5–0.25 g/L), but glucosuria and microhematuria remained. The patient was discharged with normal BP on daily prednisolone (60 mg/day for 4 weeks followed by dose tapering planned over 2 months) which resulted in complete recovery of kidney function (estimated glomerular filtration rate (eGFR) of 102 ml/min/1.73 m^2^) within 3 weeks after treatment initiation. At the last follow-up 11 weeks post-discharge from the hospital, the patient remains asymptomatic while finalizing steroid tapering with normal eGFR (106 ml/min/1.73 m^2^), isolated microhematuria, and normal BP. The time course of serum creatinine since the admission to the hospital is summarized in Figure 2.

## Discussion

This is the second case of SARS-CoV-2 associated AIN in the pediatric population and the first one reporting possible presence of SARS-CoV-2 in the kidney tissue by IHC and EM thus suggesting a possible direct involvement of the virus in the development of AIN.

Acute interstitial nephritis is an uncommon presentation of SARS-CoV-2 associated kidney disease and has been reported in a limited number of adult patients (4). It may be difficult to differentiate between secondary kidney injury and the direct role of SARS-CoV-2 in the development of intrinsic kidney disease which remains under debate (1, 2). Nevertheless, the presence of viral particles in the kidney tissue has been demonstrated by various methods, including *in situ* hybridization, RT-PCR, IHC, and immunofluorescence, while viruria has been documented in some infected patients (2).

Acute interstitial nephritis is rare in the pediatric population and has been linked to various causes, including viral (e.g., CMV or EBV) and bacterial infections with direct and indirect (immune-mediated) effects of infectious agents implicated in its development (7). A recent report by Serafinelli et al. described a similarly aged girl presenting with biopsy-proven AIN temporally (4–6 weeks after contact) related to serologically confirmed previous SARS-CoV-2 infection with complete resolution after oral prednisolone therapy. However, RT-PCR for SARS-CoV-2 in the biopsy tissue was negative (5). We have observed normalization of kidney function after the initiation of steroid therapy, similar to the aforementioned case. However, the observational nature of a single case report does not allow to make implications about therapeutic effects. Although there is no standardized guidance on the management of AIN in children's steroids are usually employed in children who fail to improve with supportive management only (7). Steroids are similarly employed in children with other post-COVID-19 complications, such as multisystem inflammatory syndrome in children (MIS-C) (8, 9).

Pediatric patients typically exhibit a milder course of COVID-19 but are prone to develop unique delayed sequelae (e.g., MIS) (6) and as such may represent an appropriate population to elucidate independent associations of SARS-CoV-2 with kidney disease. The clinical course of our patient highly resembles that reported by Serafinelli et al. (5) but providing evidence for a direct link between SARS-CoV-2 infection and AIN development is challenging. The clear temporal association, the relative rarity of AIN in children, and exclusion of other causes, such as drug toxicity and other concomitant infections, provide a basis to suspect a role of SARS-CoV-2 infection in the interstitial kidney injury in these patients.

We were able to demonstrate SARS-CoV-2 S glycoprotein by IHC in the inflammatory cell infiltrate within interstitium (potentially representing macrophages), while no positivity was detected in glomeruli or tubules. However, the detection of SARS-CoV-2 in kidney tissue has not been standardized to date. RT-PCR may be a relatively sensitive and specific tool to identify virus presence but precludes distinguishing the localization of virus (e.g., positivity from viral particles in the blood), (2) while immunofluorescence may be challenged by potential autofluorescence (10). Several studies compared IHC with *in situ* hybridization to detect viral particles in lung tissue, while one suggested poor specificity of IHC with significant observer bias, the other reported similar diagnostic performance (11, 12). Notably, in both studies, kidney biopsies from patients with SARS-CoV-2 lung injury were negative by both methods.

Even more, controversy exists for EM as a tool to detect SARS-CoV-2 particles in kidneys and other tissues. Several prior reports about viral particles in kidney biopsies received heavy criticism for falsely identifying normal cellular structures, such as clathrin-coated vesicles or multivesicular bodies, like SARS-CoV-2 (10, 13–15). Although particles in our biopsy match reported virus size (16) and exhibit heterogeneous dense dots representing nucleocapsid, the lack of being present in a membrane-bound vacuole and unclear relation to cellular structures precludes confirmatory conclusions (13, 14).

Although the identification of the virus in the kidney tissue supports the evidence of SARS-CoV-2 role, it does not inform about the mechanisms of kidney injury. We observed strong IHC positivity in some cells in the interstitial inflammatory infiltrate. Inflammatory cells, such as monocytes-macrophages, have been suggested to play a key role in the development of pathological hyperinflammatory response among patients with COVID-19. SARS-CoV-2 nucleocapsid positive macrophages have also been observed in COVID-19 patients with acute kidney tubular damage. It is, however, unclear whether this positivity may reflect phagocytosis of infected cells or direct infection of macrophages as ACE2 expression has been observed in macrophages stimulated by inflammatory stimuli (17).

Finally, it is important to highlight the time delay between the start of symptoms and kidney biopsy which was at least 6–7 weeks in our patient presenting with advanced kidney failure. Thus, it remains unknown whether viral infection could have been more extensive within kidney cells in the earlier period of the disease. The lack of IHC positivity in tubular cells, however, points toward secondary damage caused by an inflammatory response in the interstitium.

In conclusion, our case report adds to the emerging evidence of SARS-CoV-2 as a potential etiological agent of AIN in children and as such highlights the spectrum of SARS-CoV-2 manifestations in children. The demonstration of potential viral presence in kidney interstitial cells by IHC against SARS-CoV-2 spike glycoprotein suggests secondary inflammatory damage with a good response to steroid therapy. In the light of evolving virus spread and potential asymptomatic course, epidemiological history, serologic testing, and SARS-CoV-2 detection in biopsy should be considered in the work-up of children with AIN of unknown etiology. Further research on the role and mechanisms of SARS-CoV-2 mediated kidney injury and the role of inflammatory cells is needed.

## Data Availability Statement

The original contributions presented in the study are included in the article/Supplementary Material, further inquiries can be directed to the corresponding author.

## Ethics Statement

Written informed consent was obtained from the minor(s)' legal guardian/next of kin for the publication of any potentially identifiable images or data included in this article.

## Author Contributions

KA conceptualized the manuscript. JB and AL performed and interpreted histological investigations. KA and AJ collected and interpreted clinical data. All authors critically revised and approved the final version of the manuscript.

## Conflict of Interest

The authors declare that the research was conducted in the absence of any commercial or financial relationships that could be construed as a potential conflict of interest.

## Publisher's Note

All claims expressed in this article are solely those of the authors and do not necessarily represent those of their affiliated organizations, or those of the publisher, the editors and the reviewers. Any product that may be evaluated in this article, or claim that may be made by its manufacturer, is not guaranteed or endorsed by the publisher.
