# Supplementary material for: Case Report: SARS-CoV-2 Associated Acute Interstitial Nephritis in an Adolescent
Source: Front Pediatr. 2022 Apr 14;10:861539. doi: 10.3389/fped.2022.861539 (PMC9047909; doi:10.3389/fped.2022.861539)

Supplementary Material

**SARS-CoV-2 Associated Acute Interstitial Nephritis in an Adolescent**

Karolis Azukaitis^1^, Justinas Besusparis^2^, Arvydas Laurinavicius^2^, Augustina Jankauskiene^1^

^1^Clinic of Pediatrics, Institute of Clinical Medicine, Faculty of Medicine, Vilnius University, Vilnius, Lithuania.

^2^Institute of Biomedical Sciences, Faculty of Medicine, Vilnius University, Vilnius, Lithuania.

**Supplementary Figure 1.** Electron microscopy figure of SARS-CoV-2-like particles with lower magnification.


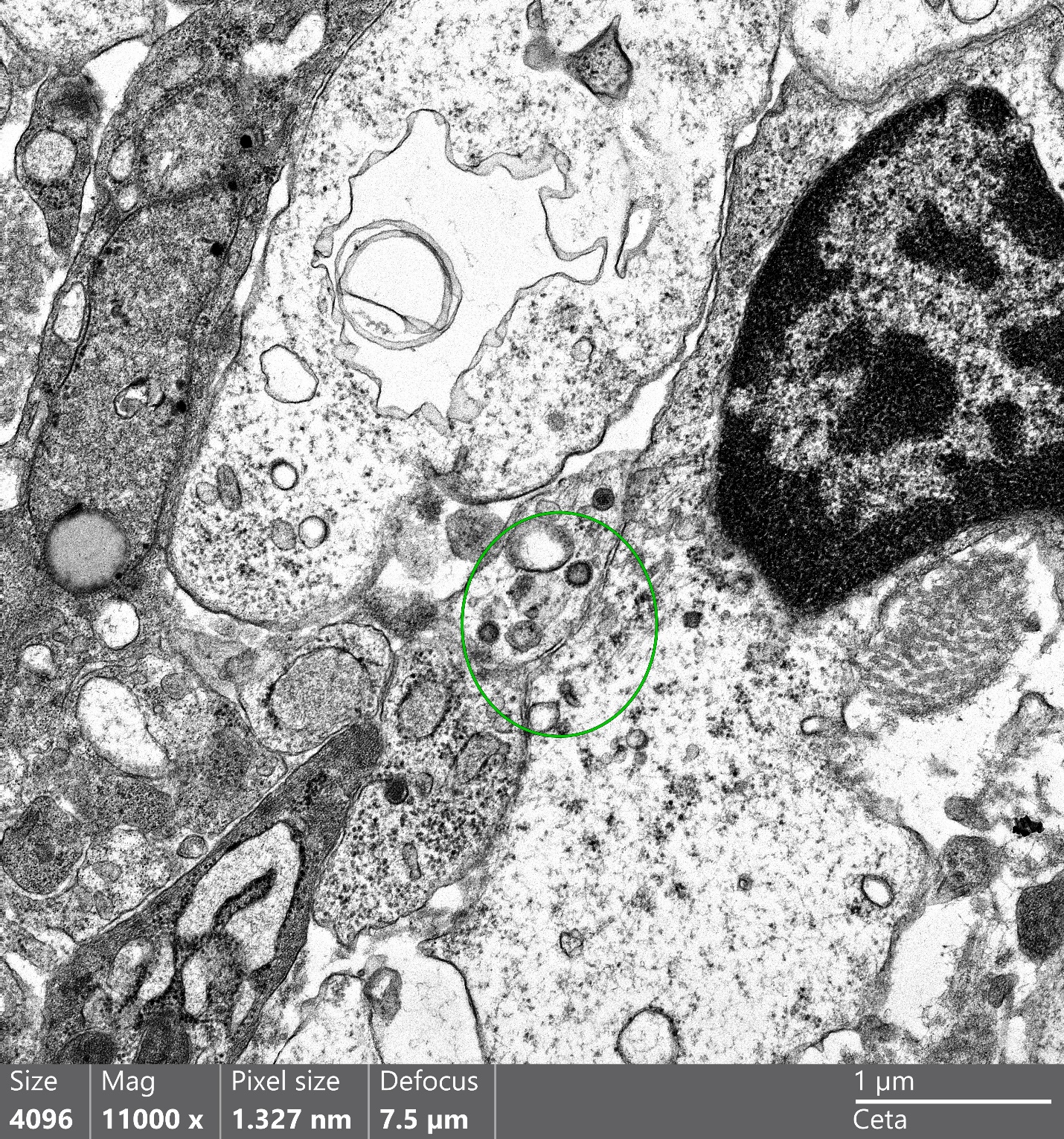


**Supplementary Figure 2A.** Positive control for SARS-CoV-2 immunohistochemistry (lung tissue).


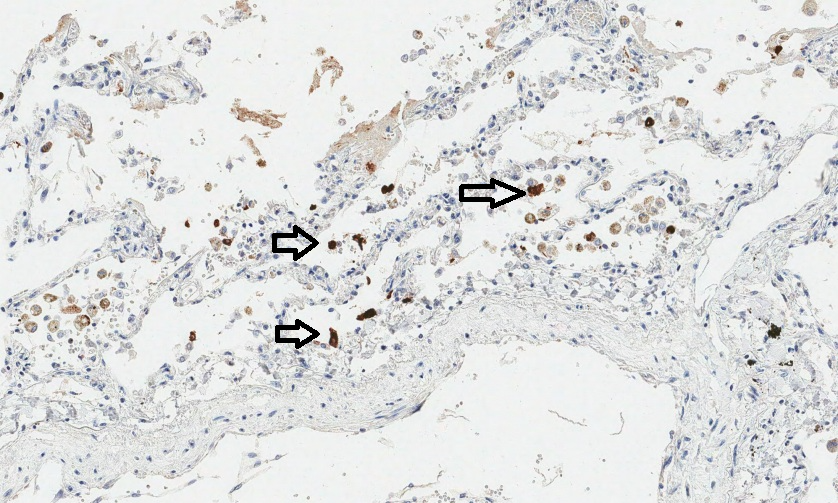


**Supplementary Figure 2B.** Negative control for SARS-CoV-2 immunohistochemistry (lung tissue).


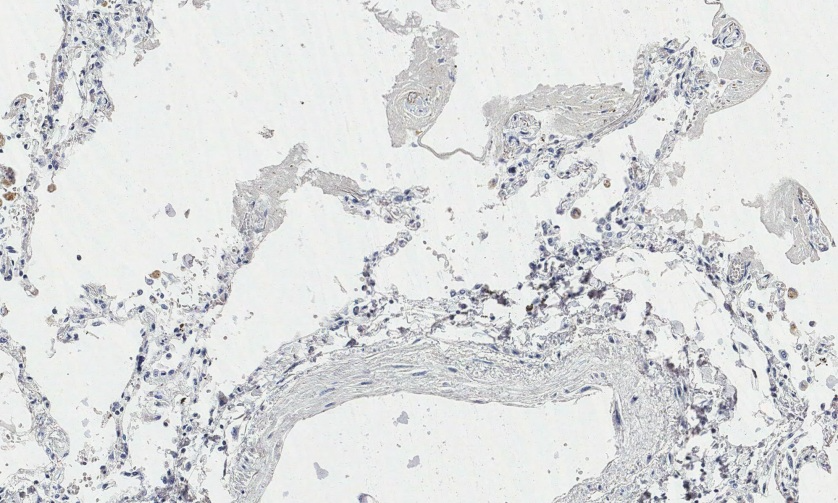


**Supplementary Figure 3A.** Negative (reagent, with omission of the primary antibody) control performed on a consecutive biopsy section


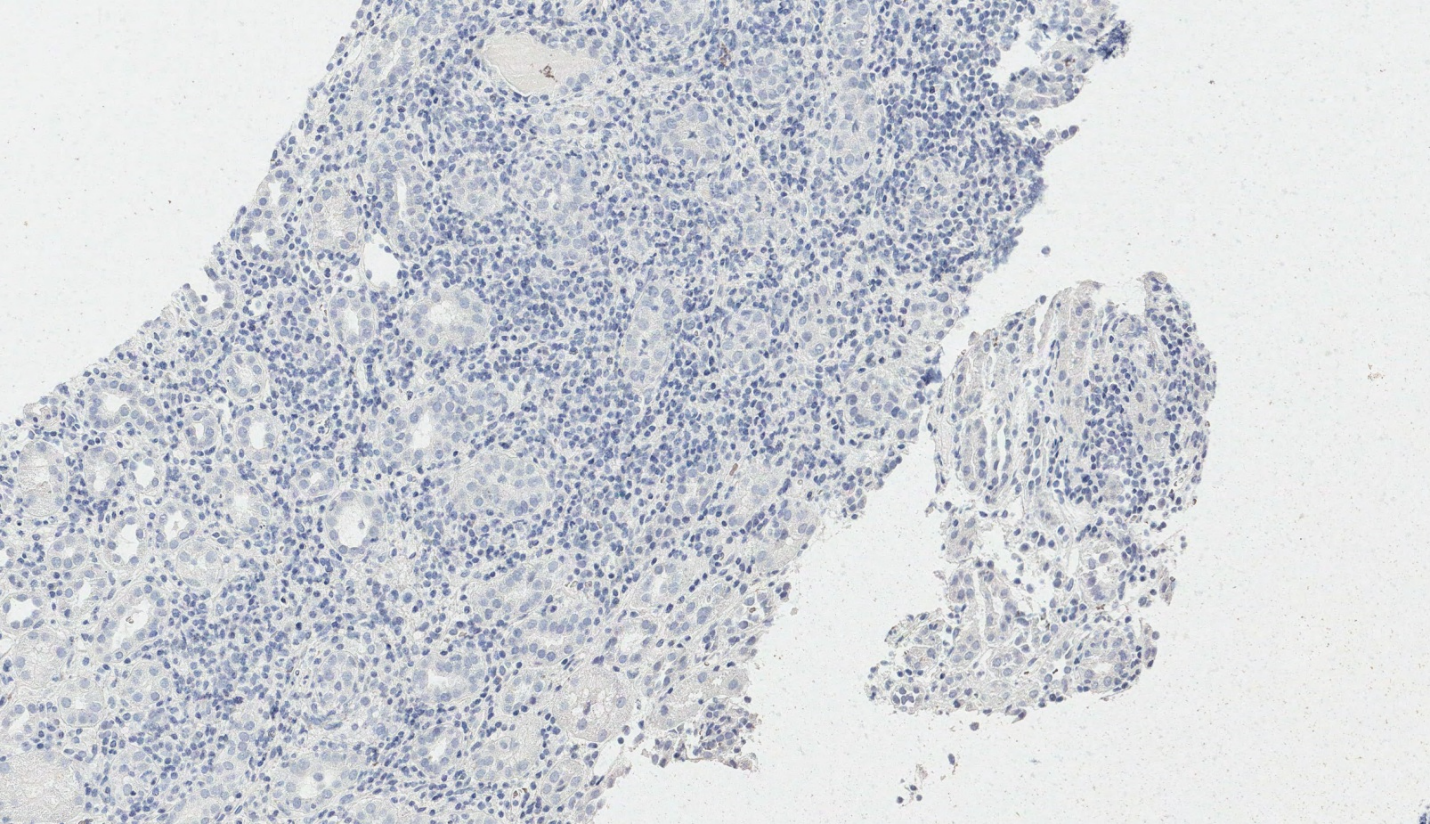


**Supplementary Figure 3B.** Mapping diagnostic SARS-CoV-2 immunohistochemistry areas with positive cells


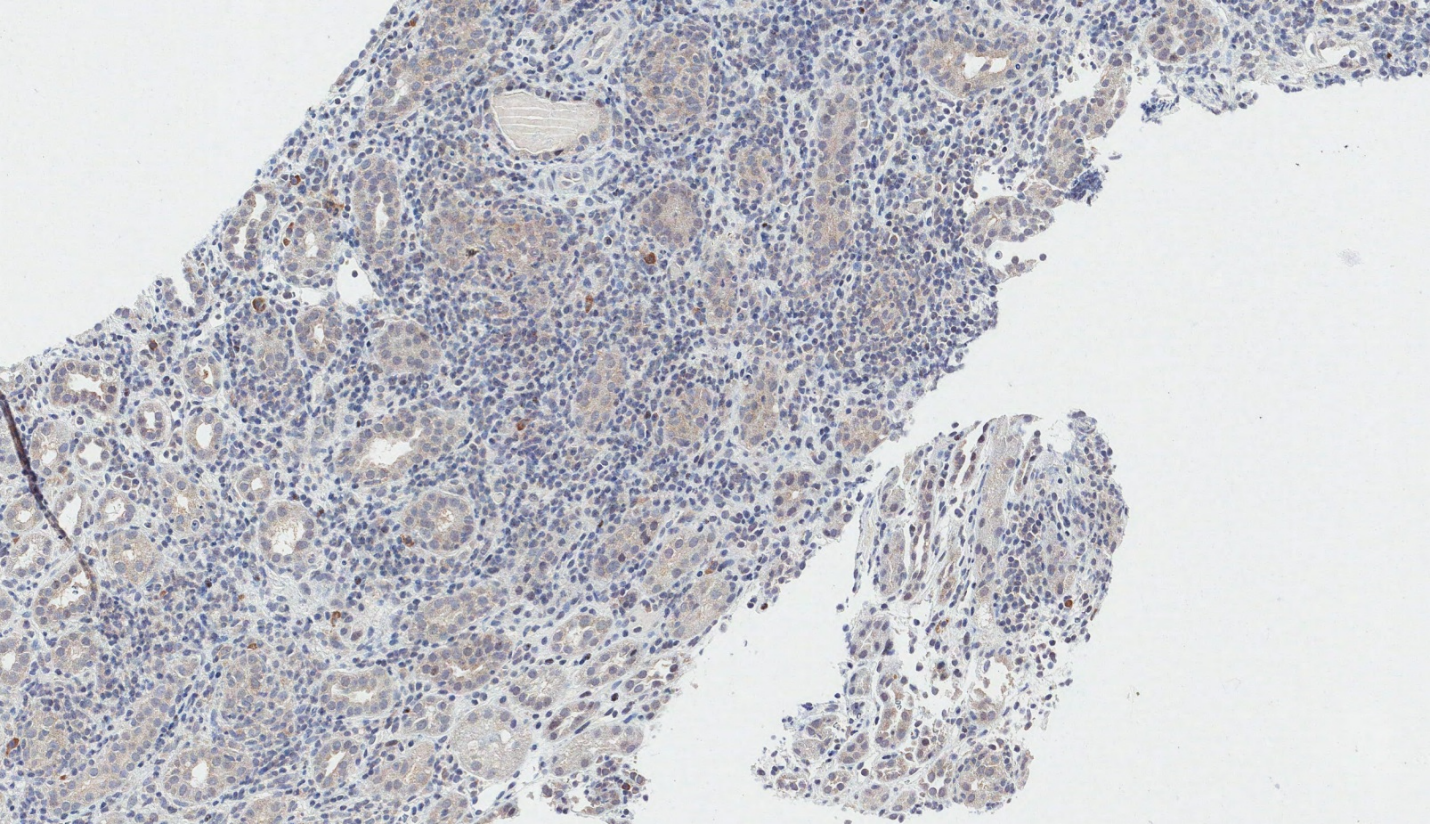


**Supplementary Figure 3C.** Negative (reagent, with omission of the primary antibody) control performed on a consecutive biopsy section


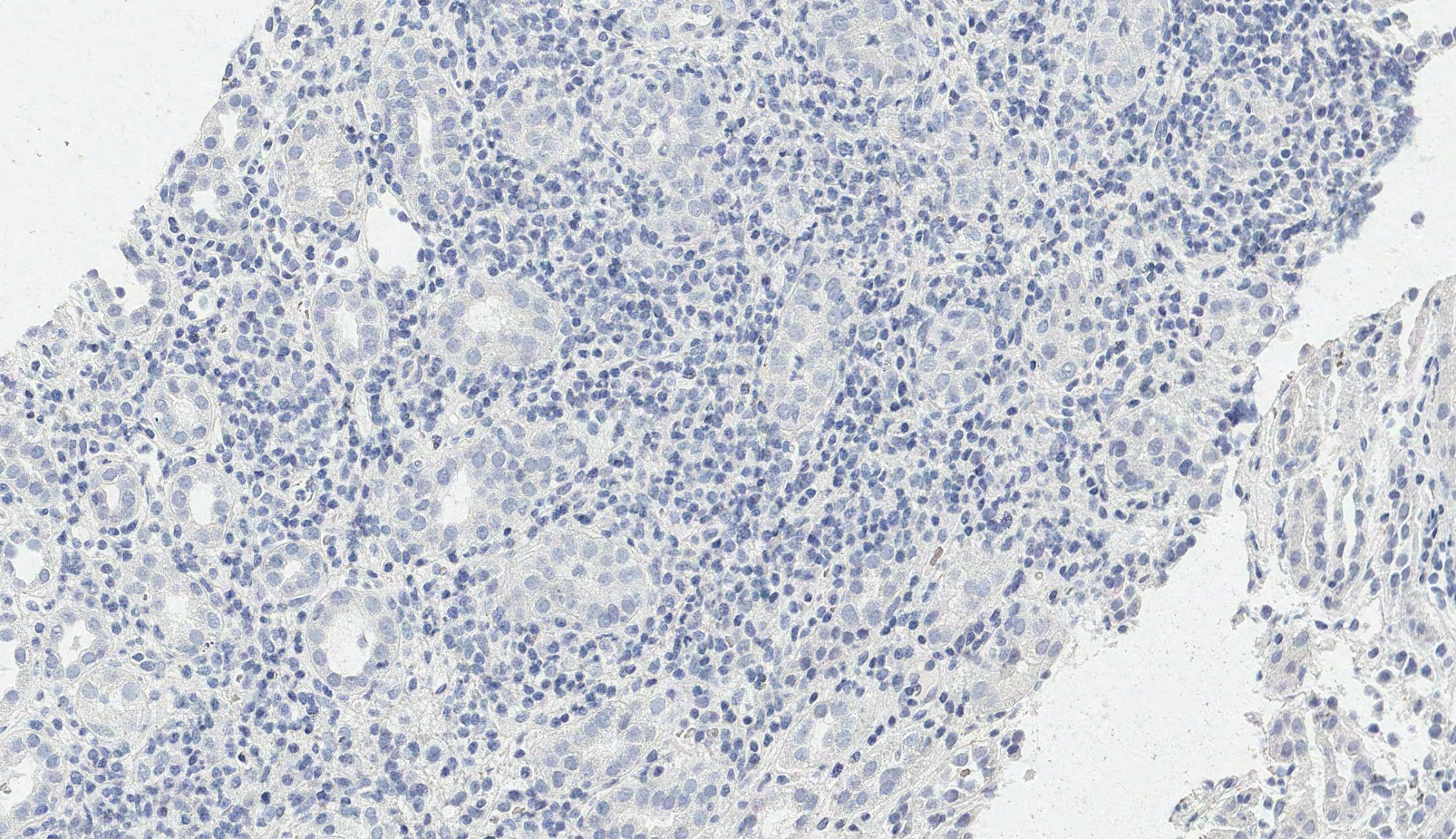


**Supplementary Figure 3D.** Mapping diagnostic SARS-CoV-2 immunohistochemistry areas with positive cells


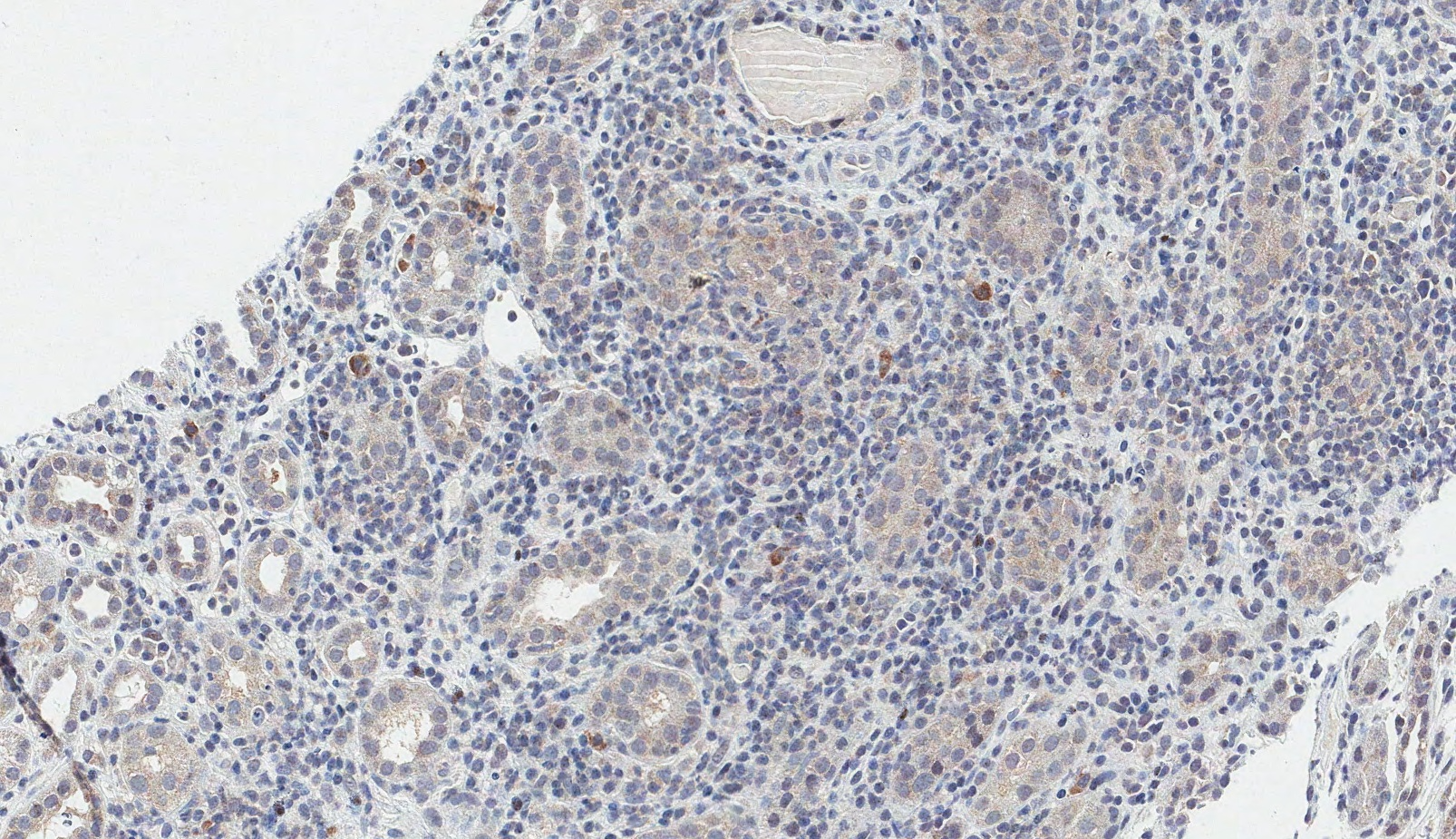


**Supplementary Figure 4**. A biopsy of a historical case of acute interstitial nephritis from other cause (dating before SARS-CoV-2 outbreak) with immunohistochemistry staining for anti-SARS-CoV-2 spike glycoprotein. The interstitial inflammatory infiltrate and other structures are completely negative.


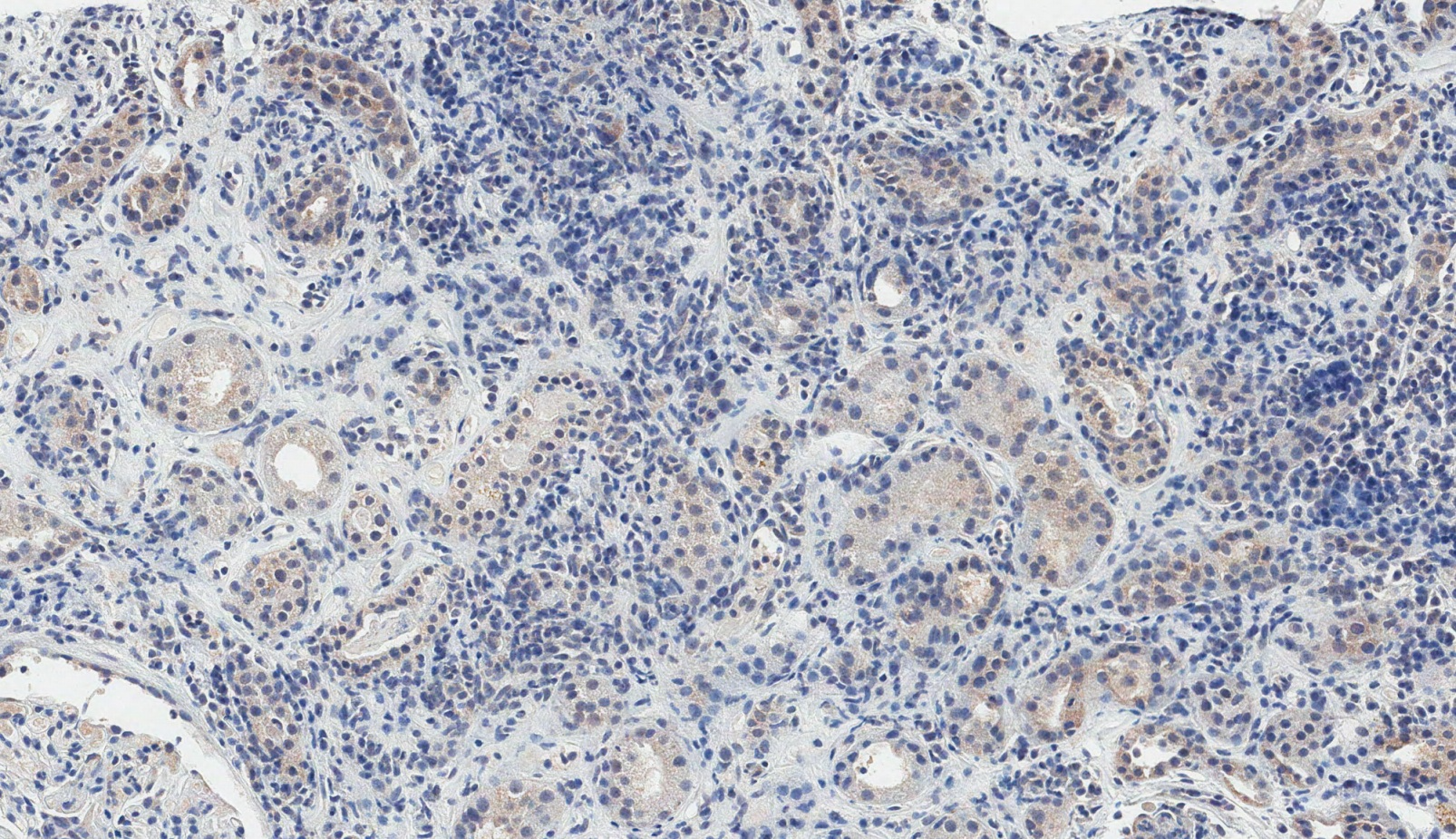

Supplement: Supplementary file 1 [file Data_Sheet_1.docx]
